# Inbreeding and fertility in Irish Wolfhounds in Sweden: 1976 to 2007

**DOI:** 10.1186/1751-0147-51-21

**Published:** 2009-05-06

**Authors:** Silvan R Urfer

**Affiliations:** 1 University of Washington Medicine Pathology, 1959 NE Pacific Street, Mailbox 357470, Seattle, Washington 98195, USA

## Abstract

**Background:**

Given that no influence of inbreeding on life expectancy could be demonstrated in Irish Wolfhounds in a previous study, it was decided to test the influence of inbreeding and other parameters on fertility in this breed.

**Methods:**

The study was based on all Irish Wolfhound litters registered in Sweden between 1976 and 2007 (n = 822 litters) as provided by the Swedish Kennel Club (SKK) and combined with a pedigree database going back to 1862. Analyses were performed using linear regression in a Generalised Linear Model and other tests in the SAS system^®^.

**Results:**

Mean number of pups per litter was 6.01 ± 2.65, with a maximum of 13. There were no significant differences in either the number of litters or the number of pups between years of birth. Males were used for breeding at a significantly earlier age than females. Mean number of litters per parent was 2.96 ± 3.14 for males and 1.59 ± 0.87 for females. No influence of Wright's inbreeding coefficients over 5, 10, 20 and 30 generations and/or Meuwissen's inbreeding coefficients on litter size was detected. In the Generalised Linear Model, highly significant, but weak (coefficient of determination (R^2^) = 0.0341) influences were found for maternal age at mating as well as maternal inbreeding measured by Wright's inbreeding coefficient over 30 generations and Meuwissen's inbreeding coefficient. Paternal inbreeding coefficients over 5, 10, 20 and 30 generations and calculated after Meuwissen, as well as maternal inbreeding coefficients over 5, 10 and 20 generations did not have significant effects on litter size.

**Conclusion:**

The low coefficient of determination (R^2^) value of the Generalised Linear Model indicates that inbreeding does not have a strong influence on fertility in Irish Wolfhounds, which is consistent with earlier results and the breed's genetic history. These results likely reflect the aforementioned genetic history and should not be extrapolated to other breeds without prior breed-specific research.

## Background

Modern purebred dogs have been created through selective inbreeding of desired phenotypes regarding both appearance and temperament, leading to frequent bottlenecks in population history. Therefore, inbreeding is a major concern in purebred dog populations, and detrimental effects of inbreeding on fitness and the incidence of hereditary diseases have been demonstrated in several breeds (e.g. [1-3]). There is currently a movement amongst both the scientific veterinary and the cynological communities to establish breeding programs with the goal of minimising inbreeding in purebred dogs. The underlying idea is that given that inbreeding has been shown to have detrimental effects that affect the well-being of dogs, the reduction of inbreeding coefficients is a matter of animal welfare [4,5].

From a population genetics point of view, inbreeding results in an increase in homozygosity, as well as the loss of alleles in a population. Inbreeding depression in a population can only occur if allelic effects are not strictly additive – some degree of dominant-recessive interaction is necessary. Two models explaining inbreeding depression can be currently found in the literature: The partial dominance model, which states that the depression is due to recessive deleterious alleles that occur more frequently in a homozygous genotype in inbred populations, and the overdominance model, which states that the inbreeding-associated decrease in heterozygosity has a negative effect in itself even in the absence of deleterious alleles [6]. Current research seems to favour the partial dominance model, although a contribution according to the overdominance model cannot be ruled out [7].

The partial dominance model proposes that inbreeding depression can be overcome by a mechanism called "purging of the genetic load": Given that deleterious recessive alleles occur in the homozygous configuration more commonly in inbred populations, selection for fitness tends to eradicate them from a population more effectively than it would in a non-inbred population [7]. Purging may be intensified after a population bottleneck if deleterious alleles are subject to selection [8,9].

In the case of Irish Wolfhounds, four distinct bottleneck phenomena resulted in particularly high inbreeding coefficients due to the small size of the effective breeding population during most of the breed's history before 1960, their geographically limited distribution and the influence of both World Wars, during which widespread food rationing made the keeping of large dogs difficult. It was already hypothesised in 1956 that the breed would no longer be subject to inbreeding depression due to its genetic history [10], and a more recent study found no influence of inbreeding on life expectancy in a population of over 1'400 Irish Wolfhounds with known lifespan out of a pedigree database of over 50'000 individuals [11].

Given that the veterinary literature states that inbreeding depression not only has a negative influence on general fitness, but also on fertility parameters such as litter size and peripartal mortality [12,13], it was decided to further test the hypothesis of a lack of inbreeding influence in Irish Wolfhounds through analysing the relationship between inbreeding coefficients and litter size in the breed. Since influences of maternal parity and season on litter size have also been described in the dog [14], the data were also analysed for a possible influence of these variables.

## Dogs, materials and methods

### Dogs

The Swedish Kennel Club (SKK) has published its registration information for Irish Wolfhounds on the internet, with information on complete registered litters going back to 1976 [15]. The SKK registers all living pups from a given litter at the latest 5 months after their birth. The absolute majority of Irish Wolfhounds in Sweden are registered with the SKK. These data were thus chosen as a means of assessing inbreeding effects on fertility on the breed. This resulted in a population of n = 5'000 dogs (2'521 males, 2'479 females) originating from 832 litters.

Pedigrees were derived from the database of the SKK and merged with an existing pedigree database going back to the beginning of modern breeding in the 1860s [11]. Litters that did not have complete pedigrees available over at least 7 generations after merging were discarded, resulting in n = 4'940 dogs (2'490 males, 2'450 females) out of 822 litters.

### Parameters Studied

We chose the number of registered pups per litter as our dependent variable and determined Wright's inbreeding coefficients [16] over 5, 10, 20 and 30 generations as well as Meuwissen's inbreeding coefficients [17] for every litter as well as every parent. Furthermore, we considered the year of birth of the litters and the ages of both sire and dam at the time of mating, as well as dam parity and season as possible influences on litter size in our population.

Number of registered pups per litter was defined as the number of pups that appear in the SKK registration database, in which no data on original litter size and peripartal mortality are made available online. Time of mating was determined by subtracting 63 days (the average gestation period of the domestic dog) from the date of birth of a litter. Ages at mating were calculated from the dates of birth of sire and dam respectively, as provided in the registration data. All ages were measured in days for statistical analysis. One litter had resulted from artificial insemination with frozen semen from a dead male; this litter was excluded from the calculation. Seasons were defined as Spring: March to May; Summer: June to August; Autumn: September to November; Winter: December to February, as used by [14].

### Software

Pedigree data were managed in Microsoft Excel^® ^2007 and Pedigree Explorer^® ^version 5.4.1FC [18] and analysed in The SAS System^® ^version 9.1.3 SP 4. Wright's inbreeding coefficients over 5, 10, 20 and 30 generations were calculated through the "Bulk COI" procedure in Pedigree Explorer, while Meuwissen's inbreeding coefficients were calculated using the meuw.exe module of the PEDIG program package [19]. Tables and bar graphs were created in Microsoft Excel 2007, while box plots were created using PROC BOXPLOT in The SAS System^®^. The box plots show minimum, 25%, 50%, 75% and maximum percentiles (red lines), as well as arithmetic means (black asterisks).

### Statistical Methods

Data were analysed for significant effects in a linear regression model using PROC GLM and PROC STEPWISE in The SAS System. A type III Sum of Squares (SS) Generalised Linear Model was used. Normality testing was performed using PROC UNIVARIATE in The SAS System, while other statistical tests (Chi-Square test, Wilcoxon rank sum test, ANOVA, Kruskal-Wallis test) were performed using PROC NPAR1WAY. A P = 0.05 was considered statistically significant.

## Results

### Population Structure

The complete data consisted of 2'521 male and 2'479 female registered Irish Wolfhound pups out of 832 registered litters. When only individuals with complete pedigree information over 7 generations were considered, the data consisted of 2'490 males and 2'450 females from 822 registered litters. Sex distribution did not significantly differ from an equal distribution model either before or after the exclusion of litters with incomplete pedigrees (χ^2 ^= 0.35, P = 0.55 before exclusion and χ^2 ^= 0.32, P = 0.57 after exclusion). The mean number of litters per sire was 2.96 ± 3.14, with a maximum of 18. The mean number of litters per dam was 1.59 ± 0.87, with a maximum of 5.

**Figure 1:**
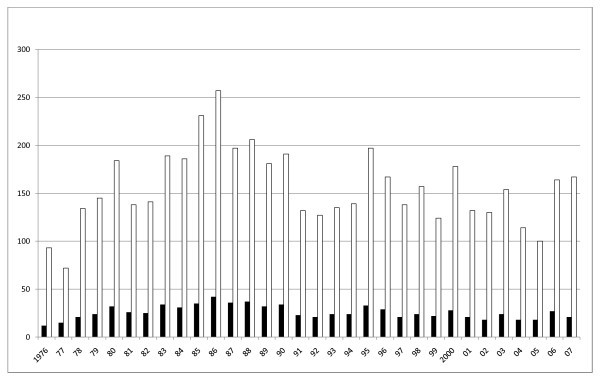
**Registered litters and pups – 1976 to 2007**. Distribution of the number of litters and the number of registered pups in the study population by year of birth. Black bars: number of litters; white bars: number of pups.

**Figure 2:**
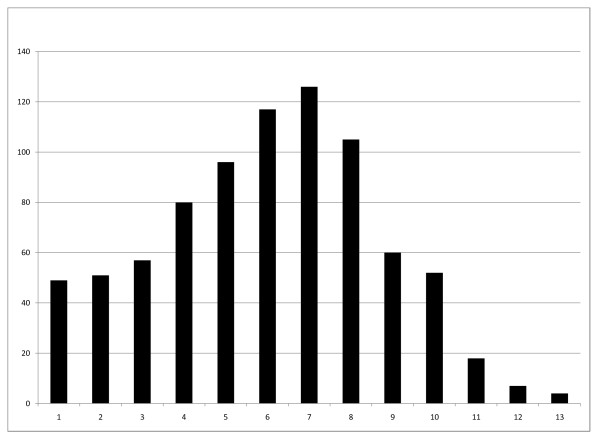
**Distribution of litter sizes (n = 822)**. Distribution of litter sizes in the study population. Horizontal axis: number of pups per litter; Vertical axis: frequency of litter size. The non-normal distribution is evident.

Figure 1 shows the distribution of litters and the number of registered pups in the studied data. Mean number of pups per litter was 6.01 ± 2.65, ranging from 1 to 13 pups per litter. Litter size distribution was non-normal (Shapiro-Wilk P < 0.0001; also see figure 2). Mean number of litters per year was 26.00 ± 7.02, ranging from 12 to 42. Mean number of registered pups per year was 156.25 ± 39.73, ranging from 72 to 257. Both of these variables were normally distributed (Shapiro-Wilk P = 0.61 and P = 0.77 respectively). There were no significant differences between either the number of litters per year (P = 0.28, ANOVA) or the number of pups per year (P = 0.66, ANOVA).

### Distribution of Inbreeding Coefficients

The results for Wright's inbreeding coefficients over 5, 10, 20 and 30 generations, as well as Meuwissen's inbreeding coefficients for every litter with complete pedigrees over at least 7 generations are reproduced in Table 1.

**Table 1:** Inbreeding coefficients

	Litters	Sires	Dams
5 Generations	0.0459 ± 0.0471	0.0602 ± 0.0582	0.0509 ± 0.0505
10 Generations	0.1559 ± 0.0555	0.1721 ± 0.0607	0.1621 ± 0.0575
20 Generations	0.2877 ± 0.0436	0.2859 ± 0.0495	0.2764 ± 0.0472
30 Generations	0.3290 ± 0.0397	0.3349 ± 0.0449	0.3245 ± 0.0433
Meuwissen	0.3331 ± 0.0407	0.3374 ± 0.0454	0.3267 ± 0.0439

Mean inbreeding coefficients ± Standard Deviation in the studied litters and their parents. Generational inbreeding coefficients were calculated using Wright's Method.

**Figure 3:**
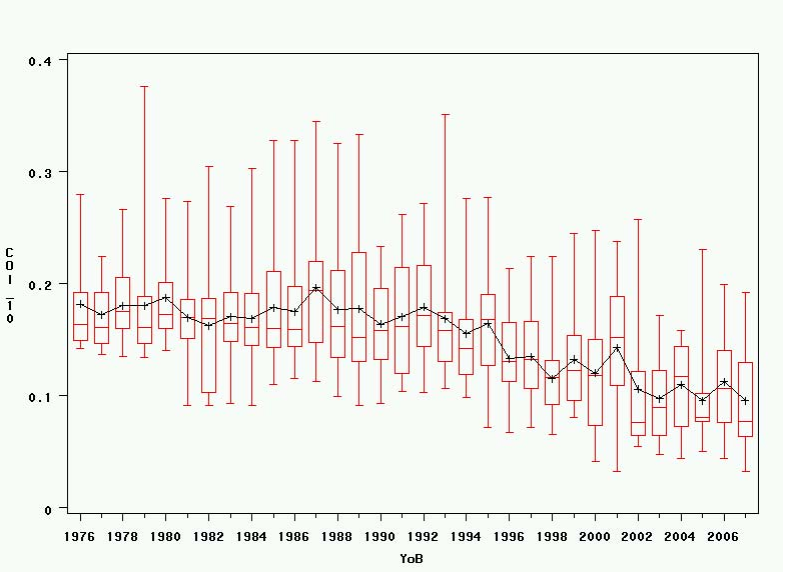
**Inbreeding over time – 10 generations**. Variations in inbreeding coefficients over time, calculated following Wright's method over 10 generations. YoB = Year of Birth; COI_10 = Wright's Inbreeding Coefficient over 10 Generations.

**Figure 4:**
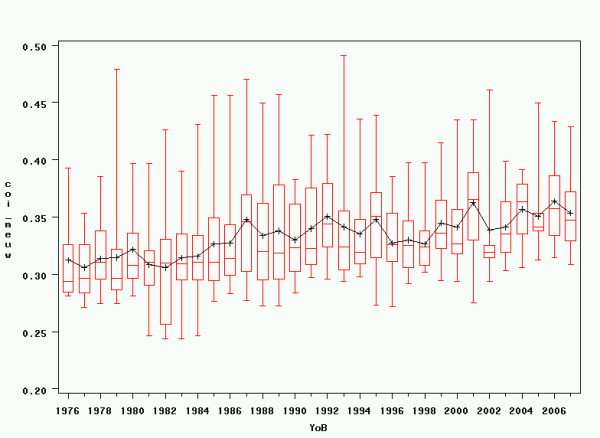
**Inbreeding over time – Meuwissen**. Variations in inbreeding coefficients over time, calculated following Meuwissen's method back to the beginning of the pedigree database. YoB = Year of Birth; COI_meuw = Meuwissen's Inbreeding Coefficient. Note the scale difference in the vertical axes between figg. 3 and 4.

The development of inbreeding coefficients over time was also analysed graphically (see figures 3 and 4). In the Generalised Linear Model, there was a significant influence of year of birth on both Wright's inbreeding coefficient over 10 generations and Meuwissen's inbreeding coefficient, with linear regression estimates of -0.003 for Wright's inbreeding coefficient over 10 generations and +0.0015 for Meuwissen's inbreeding coefficient.

### Ages at Mating, Dam Parity and Season

**Figure 5:**
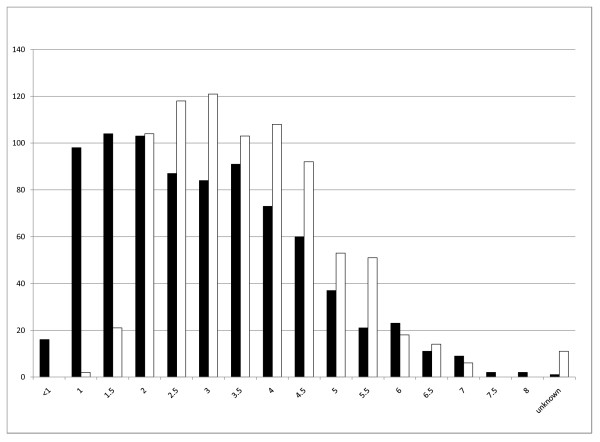
**Ages at mating**. Distribution of ages at mating in males (black) and females (white). Horizontal axis: age in years; vertical axis: number of individuals.

Mean age at mating was 1'169 ± 555 days in males and 1'396 ± 451 days in females. Ages at mating ranged from 275 to 2'948 days in males and from 512 to 2'694 days in females. This difference between the sexes was highly significant (P < 0.0001, two-sided Wilcoxon rank sum test). Ages at mating are rendered graphically in figure 5.

Given that it has been mentioned in the literature that male Irish Wolfhounds experience a more rapid decrease in semen quality than dogs do on average as they age [20,21], the age of the sire at mating was also tested separately for a possible influence on litter size. Our analysis did not show a significant influence, however (P = 0.1651, Kruskal-Wallis-Test).

Increasing maternal parity had a significant negative influence on litter size when considered by itself (P = 0.0241, Kruskal-Wallis test), but not when considered as part of the Generalised Linear Model. Results are rendered in table 2.

**Table 2:** Maternal parity and litter size

Parity	N	Mean Litter Size	SD
1	514	6.13	2.60
2	210	6.08	2.83
3	66	5.26	2.46
4	27	5.26	2.41
5	5	4.40	2.51

Maternal parity and litter size. N = number of litters; SD = Standard Deviation.

There were highly significant differences between the number of litters per season (χ^2 ^= 24.34, P < 0.0001), with the most litters being born during Spring (n = 256) and the least litters being born during Autumn (n = 156). However, no influence of season on litter size could be detected (P = 0.6634, Kruskal-Wallis test). Results are rendered in table 3.

**Table 3:** Number of litters per season

Season	Litters
Spring	256
Summer	205
Autumn	156
Winter	205

Number of litters per season. Spring = March to May; Summer = June to August; Autumn = September to November; Winter = December to February.

### Linear Regression

**Figure 6:**
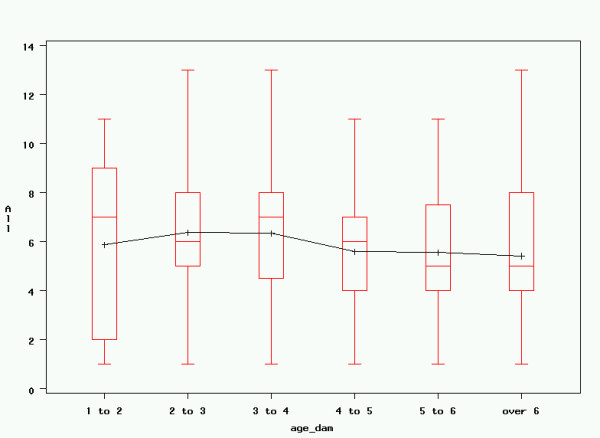
**Age of dam at mating and litter size**. Maternal age at mating and registered litter size. All: registered litter size; age_dam: maternal age at mating (years). Graphical rendering of significant effects in table 4. Although these effects are highly significant, actual influence on litter size remains low, as expressed by the low coefficient of determination (R^2 ^= 0.0341).

**Figure 7:**
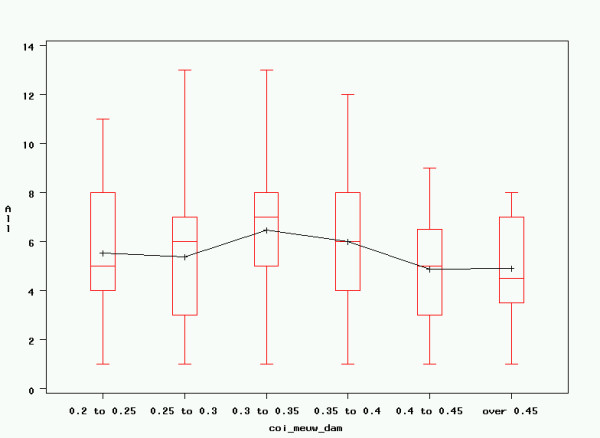
**COI (Meuw) of dam and litter size**. Maternal Meuwissen's inbreeding coefficient and registered litter size. All: registered litter size; coi_meuw_dam: maternal Meuwissen's inbreeding coefficient. Graphical rendering of significant effects in table 4. Although these effects are highly significant, actual influence on litter size remains low, as expressed by the low coefficient of determination (R^2 ^= 0.0341).

**Figure 8:**
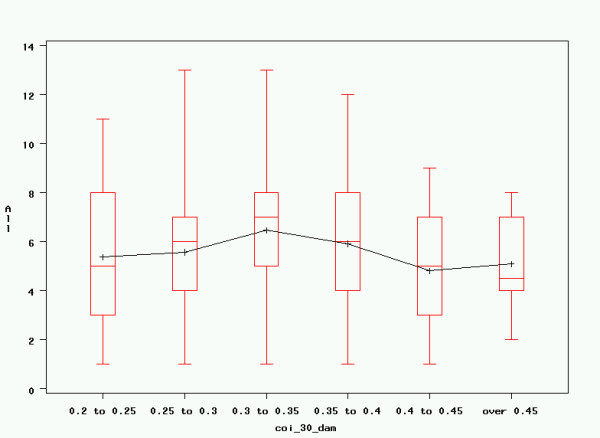
**COI (30) of dam and litter size**. Maternal Wright's inbreeding coefficient over 30 generations and litter size. All: registered litter size; coi_30_dam: maternal Wright's inbreeding coefficient over 30 generations. Graphical rendering of significant effects in table 4. Although these effects are highly significant, actual influence on litter size remains low, as expressed by the low coefficient of determination (R^2 ^= 0.0341).

Based on the variables enumerated above, linear regression was performed with the goal of constructing a model that best explained the observed variance in registered litter size. Given that normality testing showed non-normal distributions for all studied independent variables, which could not be corrected through log-transformation, a type III Sum of Squares generalised linear model (GLM) was used to establish the influence of different parameters on litter size. This resulted in significant influences of the age of the dam at mating, as well as maternal inbreeding over 30 generations and after Meuwissen; however, the coefficient of determination of the model was low (R^2 ^= 0.0341). The other variables mentioned previously did not have a significant influence on litter size in any of the models. Results are rendered in table 4 and figures 6, 7 and 8. Results for the Generalised Linear Model including all parameters are rendered in table 5.

**Table 4:** Multiple linear regression I

	F Value	P Value
Model	9.43	< 0.0001
Age of Dam	12.28	0.0005
Dam Inbreeding (30)	14.95	0.0001
Dam Inbreeding (Meuw)	14.71	0.0001

Generalised Linear Model (GLM type III Sum of Squares) of the significant parameters in our data and their influence on litter size. There are three degrees of freedom; the model has a coefficient of determination of R^2 ^= 0.0341.

**Table 5:** Multiple linear regression II

	F Value	P Value
Model	2.17	0.0010
COI 5	0.03	0.8705
COI 10	0.21	0.6487
COI 20	1.84	0.1752
COI 30	2.93	0.0873
COI meuw	2.46	0.1172
pat COI 5	1.19	0.2765
pat COI 10	0.00	0.9785
pat COI 20	0.95	0.3291
pat COI 30	2.87	0.0906
pat COI meuw	2.46	0.1171
mat COI 5	3.00	0.0838
mat COI 10	1.29	0.2570
mat COI 20	0.14	0.7113
mat COI 30	5.46	0.0197
mat COI meuw	6.99	0.0084
Parity	1.21	0.3047
Season	0.58	0.6288
Age of sire at mating	3.45	0.0637
Age of dam at mating	5.88	0.0155

Generalised Linear Model (GLM type III Sum of Squares) of all parameters in our data and their influence on litter size. There are 24 degrees of freedom; the model has a coefficient of determination of R^2 ^= 0.0627. COI 5, 10, 20, 30, meuw refer to the inbreeding coefficients of the litter, pat COI to the inbreeding coefficients of the sire, mat COI to the inbreeding coefficients of the dam.

## Discussion

While we did not find a significant effect of either a litter's own or its sire's inbreeding coefficients on registered litter size, our results demonstrate a highly significant influence of both maternal age and maternal inbreeding. However, the coefficient of determination (R^2^) of the model is low, indicating that the numeric influence of these factors is low despite their high significance. This result, combined with the fact that no influence of other inbreeding parameters (e.g. inbreeding coefficients of the litter itself and its sire) on registered litter size could be found, would seem to confirm the hypothesis that inbreeding does not have an important influence on fertility in Irish Wolfhounds.

In practice, one might call the choice of the numbers of generations used to calculate the inbreeding coefficients arbitrary (5, 10, 20, 30 generations and Meuwissen's coefficient going back to the beginning of the data). The number of considered inbreeding coefficients was kept at this level out of both practical and statistical considerations: Wright's inbreeding coefficients above 20 generations are cumbersome and time-intensive to calculate using our software, and including every possible inbreeding coefficient between two and thirty generations would have raised the risk of false positives above an acceptable level. When also considering the fact that inbreeding coefficients over different numbers of generations are not independent, testing inbreeding over every possible number of generations thus seems unnecessary.

The change in inbreeding coefficients shown in figures 3 and 4 seems striking when comparing Wright's coefficient over 10 generations and Meuwissen's coefficient. However, at least some of the decrease over 10 generations is artificial and can be explained through the exponential growth of the Irish Wolfhound breeding population that can be observed since the 1960's [11], while inbreeding coefficients calculated back to the onset of modern breeding keep increasing. Nevertheless, the fact that high inbreeding coefficients in dogs have been criticised by veterinary geneticists more recently may also have played a certain role in the recent decrease in Wright's inbreeding coefficients by motivating breeders to selectively use breeding combinations with lower inbreeding coefficients over a lower number of generations.

The study at hand is based on a database of registered pups. The studied data did not include information on how many pups in these litters died before the litters were registered, and consequently, there is no way of assessing peripartal mortality as a possible consequence of inbreeding in the data. Furthermore, the present data do not provide any means of assessing the percentage of fertile versus infertile matings. Such data are not made available online. However, the SKK litter registration forms contain fields for original litter size and peripartal mortality, and these data could be used in future research to determine whether or nor they are influenced by inbreeding.

The geographical scope of this study is limited to litters bred and registered in Sweden, which may lead to concerns of the data not being representative for the Irish Wolfhound breed in general. However, previous research has demonstrated that all Irish Wolfhounds alive worldwide during the study time can be traced back to one recent bottleneck in the 1950's [11].

The data showed a significant negative influence of maternal parity on litter size when parity was considered by itself, but this influence did not remain significant when considered as part of the Generalised Linear Model. Given that an influence of maternal parity on litter size has been described previously [14], it was decided to nevertheless include these results separately in table 2.

As opposed to reference [14], which described a significantly larger litter size for litters born during Spring, we did not find any seasonal influence on litter size, but found that a significantly increased number of litters had been born during Spring. The difference in the number of litters per season may reflect a possible influence of season on the females' sexual cycle rather than a change in other fertility parameters, which could be expected to result in seasonal changes in litter size. However, it is also possible that the Swedish breeders are planning their litters in a way that will make rearing the pups more convenient, which would seem to be easier with a litter whelped in Spring than with one whelped during Autumn due to climatic considerations.

When comparing the present results to the results of [14], however, it should be noted that the latter only found an influence of season on litter size when considering one privately owned commercial kennel, but not when considering the whole SKK database, and concluded from this that the wide variation in husbandry practices between different breeders would obscure any such influence in a large multi-kennel database. If this interpretation is correct, the same would apply to the results of the study at hand, and it is thus possible that significant seasonal effects on litter size could also exist in individual Irish Wolfhound kennels.

The relatively low average breeding age of the females in the present population can only be partially explained by the breed's comparably low life expectancy and the fact that SKK regulations generally prohibit the use of Irish Wolfhound bitches above seven years of age for breeding. It seems that in addition to these circumstances, Swedish Irish Wolfhound breeders are generally reluctant to use their bitches for breeding at a more advanced age due to their perception that this results in a higher risk of potentially deadly complications (Blom, personal communication).

Using males for breeding at a comparably young age also seems to be commonplace in the study population. This may be due to the fact that procreation tends to be less of a burden on their organism than it is in females, but may also be influenced by previous findings that male Irish Wolfhounds experience a marked decrease in semen quality and libido at a younger age than average dogs [20,21]. This could motivate the breeders to use their males at a young age, and it could also distort the influence of older males on the number of registered litters by increasing the percentage of failed matings when older males are used. While our results do not show an influence of paternal age on litter size in either the generalised linear model or the individual Kruskal-Wallis statistical test, the previously published age-related decrease in libido could also have influenced this age distribution.

## Conclusions

In view of the very low coefficient of determination (R^2^) of the Generalised Linear Model, the present data support the hypothesis that inbreeding does not play an important role among factors determining fertility in Irish Wolfhounds. More research is required to determine whether there is an inbreeding influence on fertile versus infertile matings and peripartal mortality in the breed.

In dog breeding, it is highly unusual to have data on 30 and more generation as a basis of inbreeding calculation. Therefore, and also considering the low coefficient of determination (R^2^) value of the present model, it seems unlikely that this study's findings concerning the influence of maternal inbreeding coefficients and age on registered litter size will play an important role in future Irish Wolfhound breeding practices.

The author would like to stress out that these results likely reflect the unique genetic history of Irish Wolfhounds as a breed and should not be generalised to apply to breeds in which a detrimental effect of inbreeding has been clearly demonstrated. Most breeds would not have been subject to as severe bottleneck events during their recent genetic history as Irish Wolfhounds have [11], and even in breeds that have gone through comparable bottleneck events, the elimination of inbreeding depression through purging following such events is one possible consequence, but by no means an obligatory occurrence [8,9]. Therefore, the application of the present findings to other breeds should not be attempted without first analysing similar data from these breeds.

## Competing interests

The author declares that they have no competing interests.
